# Influence of reduction quality on functional outcome and quality of life in treatment of tibial plafond fractures: a retrospective cohort study

**DOI:** 10.1186/s12891-019-2932-2

**Published:** 2019-11-13

**Authors:** Maxim Privalov, Finn Euler, Holger Keil, Benedict Swartman, Nils Beisemann, Jochen Franke, Paul Alfred Grützner, Sven Y. Vetter

**Affiliations:** MINTOS - Medical Imaging and Navigation in Trauma and Orthopaedic Surgery, BG Trauma Center Ludwigshafen at Heidelberg University Hospital, Ludwig-Guttmann-Str. 13, 67071 Ludwigshafen, Germany

**Keywords:** Fracture of Tibial plafond, Ankle joint, Lower extremity, Reduction quality, Intraoperative 3D imaging

## Abstract

**Background:**

The aim of the study was to evaluate the impact of reduction quality, using intraoperative 3D imaging, on quality of life and functional outcome in the operative treatment of tibial plafond fractures.

**Methods:**

A group of patients with tibial plafond fractures was re-examined. The operative treatment was performed between September 2001 and October 2011. The follow-up examination was at least 2 years after the final surgical procedure. Final reduction result was assessed intraoperatively using a mobile 3D C-arm. A categorization with regard to descriptive parameters as well as type and size of joint surface irregularities was performed. Follow-up results were evaluated using: Olerud and Molander (O & M) score, Short-Form-36 (SF-36) score, movement deficit, Kellgren and Lawrence grade of osteoarthritis, and pain intensity.

**Results:**

34 patients with operatively treated tibial plafond fracture could be re-examined. Reduction quality had the greatest influence on functional result measured by the O & M score (*p* = 0.001) and the PCS domain of the SF-36 score (*p* = 0.018).

Significant differences with regard to O & M score (*p* = 0.000), SF-36 score (*p* = 0.001 to *p* = 0.02; without MCS domain), movement deficit (*p* = 0.001), grade of osteoarthritis (*p* = 0.005) and pain (*p* = 0.001) could be verified under consideration of the reduction quality. The group with the anatomically more accurate reduction also showed a better result for clinical follow-up and quality of life. Furthermore, it is not the type of joint surface irregularity that is always decisive, but rather the size.

**Conclusions:**

Despite other relevant factors, it appears that reduction quality –which can be analyzed with intraoperative 3D imaging– plays the most important role in postoperative quality of life and functional outcome. Corrections should therefore be performed on joint surface irregularities with a size above 2 mm.

## Background

Tibial plafond fractures occur in approximately 5 per 100,000 people and account for about 5 to 7% of all tibial fractures [1]. More than 30% of all tibial plafond fractures are associated with high-velocity trauma, which makes operative treatment challenging due to complex fragment dislocation and severe soft tissue damage [2]. An anatomically incorrect reduction, in the sense of axial deviation or graduation of the joint surface, leads to a relevant functional limitation of joint movement and premature arthrosis [3–6]. Therefore, operative intervention with anatomical reconstruction of the joint structures is often indispensable to achieve a satisfactory clinical outcome [5, 7–9].

Intraoperative assessment of the articular surface and implant placement with conventional fluoroscopy is demanding. Studies using the cadaver model have shown that, even under optimal conditions, analysis of the joint surface and implant placement using conventional fluoroscopy may not be sufficient [10–12]. The current gold standard for preoperative planning and postoperative assessment of reduction quality and implant placement is computed tomography (CT). However, this is not standardly available for intraoperative evaluations [13].

Postoperative complications in tibial plafond fractures are well known and described in detail in the literature [14, 15]. Postoperative detection of a relevant fragment dislocation or implant misplacement can therefore lead to a surgical revision procedure in individual cases. A reliable intraoperative examination regarding the quality of reduction should make it possible to recognize and correct a malalignment during the operation.

Intraoperative 3D imaging using a mobile C-arm can be used to assess the reduction result and implant placement to identify intraoperative conditions requiring correction [16–21]. Several studies have already demonstrated that the use of intraoperative 3D imaging may lead to a relevant intraoperative revision rate of between 14.6 and 36% of the cases, despite the lack of evidence of malreduction or implant misplacement in conventional fluoroscopy [18–20, 22, 23].

Furthermore, only a few studies have investigated the functional outcome and health-related quality of life after operations due to tibial plafond fractures, but without referring to the quality of reduction [24, 25]. A study investigating the postoperative outcome of tibial plafond fractures, taking into account the reduction quality assessed by intraoperative cone-beam CT, has not yet been conducted. In most cases, the reduction quality was evaluated postoperatively [26].

The aim of the study was to investigate the influence of reduction quality in the operative treatment of tibial plafond fractures on quality of life and functional outcome while taking into account the type and size of the joint surface irregularity (assessed using intraoperative 3D imaging).

## Methods

### Establishment of the cohort

In the scope of a retrospective, monocentric study, the following exclusion criteria for a group of patients with tibial plafond fractures were applied: concomitant injuries of the same extremity, spinal injuries with neurological symptoms, polytrauma with craniocerebral trauma higher than grade I, preexisting primary and secondary osteoarthritis of the ankle joint and previously suffered injuries of the same anatomical region (e.g. ankle fractures), and postoperative complications (infection, thrombosis, compartment syndrome, flap plastic, revision in external clinics, wound healing disorder, bleeding, necrosis or amputation).

The included patients with tibial plafond fractures, classified as AO/OTA type B and C according to the preoperative CT data, formed the cohort, which was examined during the follow-up. The follow-up examination was at least 2 years after the final surgical procedure. The operative treatment was performed by experienced surgeons in a level I trauma center between September 2001 and October 2011. The final reduction result was assessed intraoperatively using a mobile 3D C-arm (cone-beam CT) (Siremobil-Iso-C-3D, Arcadis-Orbic-3D; Siemens Healthcare GmbH, Erlangen, Germany).

### Differentiation within the cohort

The collective was differentiated according to the following parameters: Age, gender, BMI, concomitant diseases, profession, type of accident (private/work-related), fractured side, type of fracture (type B/C), and concomitant injuries.

Furthermore, patients were retrospectively categorized into two groups regarding the reduction quality. The joint surface was evaluated with a dynamic inspection of the complete 3D data set. After adjusting the standard planes, “steps” in the coronal and sagittal planes were defined as deviations of the subchondral bone along the axial axis, “gaps” as voids between the fracture fragments close to the joint surface, and “defects” as frank depressions in the articular surface. The incongruencies were each measured at the point of their maximum extent. The first group was defined as reduction results with articular surface incongruencies (steps, gaps or defects) of less than or equal to 2 mm in the scan images. The second group included all patients whose incongruencies exceeded 2 mm in size of step, gap or defect. According to this classification, 15 patients were placed in Group I and 19 patients in Group II.

### Follow-up parameters

The results of the follow-up examination were assessed on the same day using the following parameters: Olerud and Molander (O & M) score, Short-Form-36 (SF-36) score, movement deficit, Kellgren and Lawrence grade of osteoarthritis, and pain intensity using a visual analogue scale (VAS).

From the SF-36 score, several scores can be derived, which are assigned to specific categories. In this case, all four domains of the score (Physical Functioning, Role Physical, Bodily Pain and General Health) were used and supplemented by the two summary scores (Physical Component Summary (PCS), Mental Component Summary (MCS)).

The severity of osteoarthritis of the ankle joint was determined using the radiographic classification by Kellgren and Lawrence. In the study, X-rays were taken for the patients with a medical indication necessitating this and who gave their consent. In two patients, no X-ray was taken due to the absence of at least one of the two conditions mentioned.

The range of motion of both ankle joints was measured with a goniometer applying the neutral-zero method and subsequently compared to the healthy contralateral side by forming differences to determine any deficit.

Furthermore, the follow-up included the current intensity of the pain in the affected region that was examined on the VAS.

### Statistical analyses

Group-specific analyses, correlation analyses and multivariate linear regression analyses were performed using the dataset collected. The statistical analysis was carried out using IBM SPSS Statistics 21 (IBM Corporation, Armonk) and Microsoft Excel 2010 (Microsoft Corporation, Redmond).

## Results

### Descriptive characteristics of the cohort

A total of 34 patients with operatively treated tibial plafond fracture could be re-examined. The average age of the study group, consisting of 8 women and 26 men, was 44.6 years at the time of surgery (SD: 11.87, range: 21–64). Patients averaged 26.8 kg/m^2^ in BMI (SD: 3.46, range: 20.96–36.51). The average period between surgery and follow-up was 64 months (SD: 31.79, range: 24–131).

Referring to the AO/OTA Classification, 20 of the patients had type B fractures and 14 suffered from type C fractures. In Group I there were 11 type B fractures and 4 type C fractures. In Group II there were 9 type B fractures and 10 type C fractures.

### Olerud & Molander and short-Form-36

On average, the patients surveyed scored 69 points (SD: 24.79, range: 10–100) in the O & M score. The comparison of both groups according to the reduction quality is shown in Table 1 below. In the SF-36 survey, the PCS averaged 48 points (SD: 10.6, range: 25.22–60.77) and the MCS 51 points (SD: 9.58, range: 27.22–64.83). The PCS score distribution in the two groups is shown in Table 2.

**Table 1:** Descriptive statistics for the Olerud and Molander scores with comparison of the groups (Group I = good reduction; Group II = suboptimal reduction). The values correspond to the scores achieved

Reduction quality	Mean	Median	Standard deviation	Min.	Max.	25%-Percentile	75%-Percentile
Group I	88.00	95.00	15.09	60.00	100.00	82.50	100.00
Group II	54.21	55.00	20.43	10.00	100.00	45.00	65.00

**Table 2:** Descriptive statistics with score distribution for the Physical Component Summary (SF-36) in both groups (Group I = good reduction; Group II = suboptimal reduction). The values correspond to the scores achieved

Reduction quality	Mean	Median	Standard deviation	Min.	Max.	25%-Percentile	75%-Percentile
Group I	53.97	57.93	7.64	35.52	60.77	48.17	59.40
Group II	43.73	43.12	10.58	25.22	60.61	34.91	51.97

### Grade of osteoarthritis

**Fig. 1:**
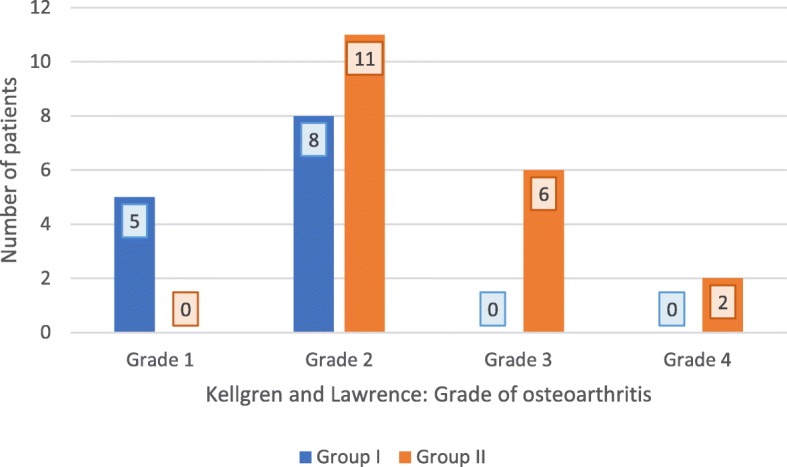
Distribution of patients in terms of grade of osteoarthritis according to Kellgren and Lawrence. Direct comparison of the number of patients in each group (Group I = good reduction; Group II = suboptimal reduction) using a bar chart

Figure 1 shows the categorization of the two patient groups according to their grade of osteoarthritis classified by Kellgren and Lawrence.

### Range of motion

**Fig. 2:**
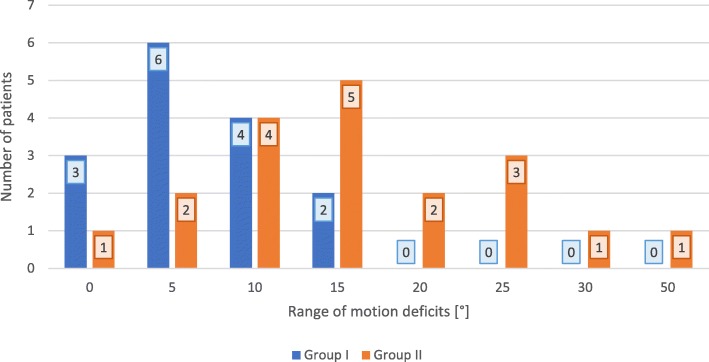
Distribution of patients in both groups for range of motion deficits. Number of patients from each group are assigned to the respective movement deficit in degrees [°] represented as bar chart

Figure 2 shows the number of patients with the deficit of range of motion depending on the group affiliation for the reduction result.

### Pain intensity

The mean value for the intensity of pain, according to the VAS, was 2.88 and the median was 2.5 (SD: 2.57, range: 0–8). The illustration of the group-specific results for the VAS is shown in Table 3.

**Table 3:** Descriptive statistics for pain intensity using the visual analogue scale with a comparison of the two groups (Group I = good reduction; Group II = suboptimal reduction). The values of pain intensity are on a scale from 1 to 10

Reduction quality	Mean	Median	Standard deviation	Min.	Max.	25%-Percentile	75%-Percentile
Group I	1.33	1.00	1.88	0.00	7.00	0.00	2.00
Group II	4.11	5.00	2.40	0.00	8.00	3.00	5.50

### Group-specific comparison concerning reduction quality

The O & M score could be significantly influenced by the reduction quality (*p* = 0.000): The mean difference was 34 points (SE: 6.32).

Significant association of SF-36 score with reduction quality could also be observed (*p* = 0.001 to *p* = 0.02; without MCS domain): In the comparison of the PCS domain, the mean difference amounted to 10 points (*p* = 0.003; SE: 3.25). There were no significant differences with regard to the MCS domain of the SF-36 score (*p* = 0.142).

There were significant differences of movement deficit when reduction quality was compared (*p* = 0.001): The mean ranks of the good reduction group were lower (11.50°) than the mean ranks of the suboptimal reduction group (22.24°).

Significant deviation in pain level, captured by VAS, depending on reduction quality (*p* = 0.001) was found: The mean difference was 2.77 (SE: 0.76).

A significantly different distribution between Group I and Group II concerning the grade of osteoarthritis (*p* = 0.005) could be seen (Fig. 1).

In summary, the group with the anatomically more accurate reduction showed a better result in terms of clinical follow-up and quality of life except for the MCS domain of the SF-36.

### Group-specific comparison concerning descriptive parameters

No significant differences could be found concerning age (*p* = 0.836), sex (*p* = 0.231), BMI (*p* = 0.151), type of fracture (*p* = 0.127) or period between surgery and follow-up (*p* = 0.996) in the groups differentiated according to reduction quality.

Significant distribution differences were observed with regard to nicotine abuse (*p* = 0.002), profession with heavy physical work (*p* = 0.014) and concomitant injuries (*p* = 0.004), whereby these were predominantly found in the suboptimal reduction group (Group II).

Nicotine consumption had a significant influence on the O & M score (*p* = 0.003) and the profession category with heavy physical stress showed a significant influence on the movement deficit (*p* = 0.000), which in each case was associated with a worse outcome. The degree of concomitant injuries correlated negatively with the O & M score (*p* = 0.009, *r* = − 0.442) as well as with the Role Physical domain of SF-36 (*p* = 0.042, *r* = − 0.351) and was associated with a worse result in the range of motion (*p* = 0.008, *r* = 0.446) along with the pain intensity (*p* = 0.005, *r* = 0.472). The other parameters remained unaffected.

A correlation between the period for the follow-up examination and the individual examination parameters could not be observed either (*p* = 0.200–0.937, *r* = 0.160).

### Group-specific comparison concerning type of articular surface irregularities

Table 4 lists the descriptive statistics regarding the O & M scores depending on the type of articular surface irregularity. Only the group of patients with steps differed significantly from those with the combination of gaps and defects (*p* = 0.034). None of the other groups provided significant differences regarding the comparison of their mean values in the O & M scores.

**Table 4:** Descriptive statistics of the Olerud and Molander Score with distribution regarding the type of single and combined joint surface irregularities. The values correspond to the scores achieved

Type of joint surface irregularities	N	Mean	Standard deviation	Min.	Max.
Step	4	97.50	2.89	95.00	100.00
Gap	9	68.89	24.47	40.00	100.00
Defect	2	60.00	21.21	45.00	75.00
Step + gap	9	72.22	17.34	45.00	95.00
Step + defect	4	51.25	18.87	25.00	65.00
Gap + defect	4	41.25	24.28	10.00	65.00
None	2	100.00	0.00	100.00	100.00
Total	34	69.12	24.79	10.00	100.00

When parameters (SF-36, range of motion, arthrosis, VAS) were considered, no significant differences could be found concerning the different types of joint surface irregularities (*p* = 0.076–0.234).

### Group-specific comparison concerning size of articular surface irregularities

The width of the gaps ranged from 0 to 8.3 mm (SD: 1.74), the range of defects was 0 to 9 mm (SD: 2.7) and the steps varied from 0 to 4.7 mm (1.27).

The correlations between the O & M Score and the step, gap, and defect sizes revealed the results listed in Table 5, whereby only the defect size correlated significantly (*p* = 0.005; *r* = − 0.470) with the O & M Score. The score decreased with increasing defect size.

**Table 5:** Pearson correlation analysis between the Orelud and Molander Score and the size of the different joint surface irregularities

		Step size	Gap size	Defect size
Olerud & Molander Score	Pearson correlation coefficient	−0.230	−0.201	−0.470
	Sig. (2-tailed)	0.896	0.254	0.005
	N	34	34	34

The correlation analysis according to Pearson regarding the size of the specific joint surface irregularities and the six domains of the SF-36 score did not yield any significant results.

The irregularity sizes correlated with the movement deficits. Only the defect size demonstrated significant results in the correlation analysis with the extension deficit (*p* = 0.038; *r* = 0.358), the flexion deficit (*p* = 0.041; *r* = 0.353), and the total deficit (*p* = 0.013; *r* = 0.420). The Spearman coefficients were positive.

The step size (*p* = 0.807) and defect size (*p* = 0.084) did not correlate significantly with the grade of osteoarthritis. However, the gap size correlated significantly (*p* = 0.035) and the Spearman coefficient was positive. A larger gap in the articular surface resulted in a higher grade of osteoarthritis.

The VAS did not correlate significantly with either the step size or the gap size. The defect size, however, showed a significant result (*p* = 0.012). The Pearson coefficient was positive (*r* = 0.425). It could therefore be concluded that larger defects were associated with higher values on the VAS.

### Most important influencing factor related to the outcome

According to the multivariate linear regression analyses of this study, the reduction quality had the greatest influence on the functional result after operatively treated tibial plafond fracture determined by the O & M score (*p* = 0.001) and the PCS domain of the SF-36 score (*p* = 0.018).

## Discussion

The operative treatment of intra-articular tibial plafond fractures remains difficult even for the experienced trauma surgeon, since the intraoperative assessment of the tibial joint surface and the implant placement using conventional fluoroscopy is limited [27–30]. The aim of this study was to evaluate the impact and benefit of reduction quality, using intraoperative 3D imaging criteria, in terms of postoperative outcome on the follow-up of 34 patients with tibial plafond fractures type B and C according to the AO classification.

Previous publications with a smaller number of cases showed that intraoperative 3D imaging may be beneficial for the operative treatment of tibial plafond fractures [16, 18–20]. Studies dealing with functional outcome and health-related quality of life after operations due to tibial plafond fractures have already been published. In a comparison of hybrid external fixation versus two-stage management with final plate fixation, Cisneros et al. describe persistent pain (Numerical Rating Scale 2.64–3.1) in 31 patients with tibial plafond fractures at a follow-up after 2 years. In the context of a follow-up examination of 21 patients after a median period of 3 years, Stengel et al. showed that the functional prognosis in SF-36, especially considering the PCS and MCS, and the associated quality of life of tibial plafond fractures remain unsatisfactory despite clear improvements in surgical management. Compared with the population-based norm, the function and range of motion of the affected ankle joint were significantly reduced [24–26]. Similar results could also be observed in our investigations using the VAS, as well as the clear deviation of the PCS in SF-36 from the normal population. Investigations evaluating the postoperative outcome of tibial plafond fractures in conjunction with anatomical reduction using intraoperative 3D imaging criteria have not yet been published.

Previous studies focused on whether an anatomically correct reduction of the tibial joint surface in tibial plafond fractures results in a prognostic difference in patient outcomes. These studies concluded that remaining joint gaps or steps of more than 2 mm after the reduction and axial deviations in the frontal or sagittal plane of more than 5 degrees could lead to poorer clinical results and higher osteoarthritis rates [5, 31–35]. Resch et al. could even demonstrate that a postoperative incongruity of the articular surface is followed by heavier arthrosis than a comparable incongruity after conservative treatment [33]. De-las-Heras-Romero et al. analyzed the impact of intra-articular tibial plafond fractures and the predictive factors on patients’ quality of life. They had already revealed that fracture severity, reduction quality, and arthrosis were the main prognostic factors, and showed that the SF-36 scores (PCS 54.8; MCS 63.3) and the Olerud and Molander score (60.1) are significantly lower than in the age-matched general population [26]. Our investigations also provided a similar result. Patients with poorer reduction results, in terms of gaps, steps, and articular surface irregularities of more than 2 mm, also showed significantly worse results in terms of quality of life and clinical-functional outcome. Moreover, the correlation analysis between the size of the irregularity and the movement deficit showed positive Spearman coefficients, which is why it could be concluded that large defects were associated with large movement deficits. Secondly, regression analyses, regardless of group affiliation, confirmed that the reduction result is the most important factor affecting postoperative outcome.

The investigations of Falzarano et al. provided a similar result: In a comparison of the different surgical techniques for the treatment of tibial plafond fractures, they showed that incorrect reduction can lead to changes in the sagittal balance line for foot loading and pace training, regardless of the type of surgical procedure [36]. Therefore, an anatomically correct reduction and restoration of the joint surface is desirable. On the one hand, the choice of the optimal operative procedure is essential. For instance, the study by Bisaccia et al. showed that the locked plate is more advantageous than the intramedullary nail in the treatment of distal extraarticular tibial fractures in terms of an anatomically correct reduction of the fracture with a lower rate of non-unions [37]. On the other hand, intraoperative 3D imaging may already provide additional information during the initial operative procedure and may therefore enable the surgeon to intraoperatively perform corrections of the reduction and implant [18–20, 22, 23]. This can potentially avoid the need for revision surgery, decrease the associated perioperative risks for the patient, and at the same time positively influence the long-term outcome.

Smoking as a risk factor correlated significantly with the Olerud and Molander score. Nicotine consumption led to a lower score. Since this risk factor with a significant distribution difference was found predominantly in Group II, nicotine consumption, in addition to reduction quality, also plays an important role in follow-up outcome. The negative effect of nicotine consumption on osteogenesis and fracture healing has already been well demonstrated in vitro and in vivo test series [38, 39]. Furthermore, a recent study showed that nicotine consumption influences pain perception and therefore smokers are dependent on significantly more analgesics postoperatively [40]. Other studies reported smoking as a predictive factor for musculoskeletal complaints, defined as having pain and/or stiffness in muscles and joints [41].

In addition to smoking, physical stress at work played a decisive role in both groups and had a negative influence on the outcome. A meta-analysis of distal radius fractures showed a similar result, but a correlation could also be demonstrated in the surgical treatment of proximal humerus fractures [42, 43].

Concomitant injuries in the area of the affected body region leading to worse clinical results can be confirmed for almost every type of fracture. However, for concomitant injuries in other body regions it could only be shown that these usually lead to a longer hospital stay, which is associated with a higher mortality rate in elderly patients in particular. Generally, this had no influence on the outcome of the examined injury [44, 45]. In contrast, in our study, the concomitant injuries, secondary to the reduction result, showed a negative influence on four of the evaluated outcome parameters.

The study has several limitations. The absolute number of 34 participants was quite low and so allows only a limited statement about the overall population. Furthermore, the resulting high range made the statistical evaluation of some results difficult. Nevertheless, given the low incidence of the type of injury, the fact that type B and C fractures are very rare, and the long follow-up period, the number of patients examined compared to other studies is actually very high.

## Conclusions

In conclusion, the established reduction criteria in intraoperative 3D imaging appear to have the highest impact on postoperative quality of life and functional outcome despite other relevant factors such as nicotine consumption, concomitant injuries or profession with heavy physical stress.

Furthermore, it is not the type of joint surface irregularity that is always decisive, but rather the size. This should be considered in the reduction analysis and corrected if necessary, especially if the surface irregularity is above 2 mm.

## Data Availability

The datasets used and analyzed during the current study are available from the corresponding author on request.

## Abbreviations

ANOVA: Analysis of variance
BMI: Body mass index
CT: Computed tomography
MCS: Mental Component Summary
O & M: Olerud and Molander
PCS: Physical Component Summary
SD: Standard deviation
SE: Standard error
SF-36: Short-Form-36
VAS: Visual analogue scale
